# Comparative Digital Estrogen Receptor Alpha (ERα) Expression Analysis in Benign and Malignant Prostate Tissue of Men and Dogs

**DOI:** 10.1002/pros.70111

**Published:** 2025-12-23

**Authors:** Jennifer Lothion‐Roy, Leonore Aeschlimann, Lea Anna Hiller, Sven Rottenberg, Nigel P. Mongan, Catrin S. Rutland, Emad Rakha, Alexander Dean, Mark A. Rubin, Simone de Brot

**Affiliations:** ^1^ Biodiscovery Institute, University of Nottingham University Park Nottingham UK; ^2^ School of Veterinary Medicine and Science, University of Nottingham Sutton Bonington campus Loughborough UK; ^3^ COMPATH, Institute of Animal Pathology, Vetsuisse Faculty University of Bern Bern CH Switzerland; ^4^ Bern Center for Precision Medicine University of Bern Bern CH Switzerland; ^5^ Department of Pharmacology Weill Cornell Medicine New York NY USA; ^6^ School of Medicine, University of Nottingham University Park Nottingham UK; ^7^ Pathology Department Hamad Medical Corporation Doha Qatar; ^8^ Department for Biomedical Research, Vetsuisse Faculty University of Bern Bern Switzerland

**Keywords:** canine model, digital pathology, immunohistochemistry, prostate cancer

## Abstract

**Background:**

The dog is the only large mammal, other than humans, that commonly develops spontaneous prostate cancer (PCa) and is, therefore, considered a valuable model for comparative studies. Estrogens are critical for normal prostate development and contribute to prostatic carcinogenesis in men. The number of transgender women undergoing male to female transition involving exogenous estrogen treatment and surgical or chemical castration has increased markedly in recent years. Few studies have evaluated estrogen receptor α (ERα) expression in benign and malignant canine prostatic tissue, and comparative data between dogs and men are currently lacking. This study analyzed and compared the spatial distribution and level of ERα expression in the benign and malignant prostatic tissue of men and dogs using immunohistochemistry (IHC) and assessed the suitability of dogs as a model to further understand the role of ERα in human PCa.

**Methods:**

Formalin‐fixed paraffin‐embedded (FFPE) human (*n* = 146) and canine (*n* = 61) prostatic tissue specimens were analyzed immunohistochemically for ERα expression using a monoclonal anti‐human ERα antibody, previously validated for cross‐reactivity with canine tissue. Nuclear staining was digitally quantified with Visiopharm software. Tissue segmentation allowed separate analyses of ERα expression patterns in both epithelial and stromal compartments.

**Results:**

ERα expression was present in the stroma of both non‐malignant and neoplastic prostatic tissue in men and dogs. Both non‐malignant and malignant human glandular epithelium was consistently negative for ERα. In contrast, benign glandular epithelium in sexually intact dogs expressed ERα, showing weak but consistent immunolabeling. Malignant transformation in canine glands was associated with a reduction of ERα expression compared with benign tissue.

Similarly, non‐secretory glands in premature and atrophic (both castration‐induced and age‐related) canine prostates exhibited very low levels of ERα expression. Higher stromal ERα expression was observed in premature canine prostatic tissue when compared with mature, confirming the relevance of ERα in prostate development.

**Conclusions:**

Malignant glandular epithelium lacked ERα expression in both dogs and men, with a notable shift from epithelial to stromal ERα expression in dogs during neoplastic transformation. Unlike in men, benign canine glands show diffuse ERα expression, whereas premature and atrophic glands display very low ERα levels. The observed differences in ERα expression across prostate tissue types in the dog —premature, normal, atrophic, and tumor—warrant further investigation to provide a clearer understanding of the role of ERα in PCa progression, particularly in castration‐resistant cases. Such insights gained from the canine disease model may also help guide screening and management strategies for the growing population of young transgender women undergoing estrogen therapy and orchiectomy.

## 1 Introduction

Prostate cancer (PCa) is the most commonly diagnosed male cancer type in the Western Hemisphere and continues to prove a major clinical challenge [1, 2]. Estrogens and their alpha receptor (ERα) are essential for the normal development and performance of the male reproductive tract [3, 4, 5, 6], but have also been associated with the development and progression of PCa [7, 8, 9, 10].

Upregulated ERα expression has been observed with malignant transformation in the prostate and following ADT [11]. This suggested oncogenic role of ERα in the prostate is further supported by the finding that ERα‐knockout mice do not develop PCa in experimental carcinogenesis following testosterone and estradiol treatments in contrast to their wild type counterparts [12]. Despite a number of studies examining the role of estrogens and ERα in PCa, particularly in rodent models [13, 14, 15, 16, 17], the use of anti‐estrogen therapies in PCa patients has thus far only been trialed on a small scale and with variable results [18, 19, 20]. The role of ERα in human PCa therefore warrants further attention but remains poorly understood.

In fetal and early neonatal life, ERα expression is widespread throughout all prostatic tissue, reflecting its role in organogenesis [21, 22]. In normal adult human prostate tissue, ERα expression is largely limited to stromal cells and subsets of proliferative androgen independent basal cells, with glandular epithelial cells generally ERα negative [11, 23, 24, 25, 26].

Among large mammals, dogs are the only species to spontaneously develop PCa with some regularity [27, 28, 29, 30]. Although less common than in humans (incidence: 0.3‐0.6%) [31, 32], canine PCa typically presents during older age [33, 34] and is often aggressive, with metastasis already present at the point of diagnosis in ~ 40% of cases [27, 34, 35]. The metastatic pattern closely mirrors that observed in men [34, 35, 36, 37]. Additionally, the anatomy and embryological origin of the canine prostate more closely resemble those of humans than the rodent models commonly used to date [38]. Given that they also share our living environment, it has been suggested that dogs may serve as effective models for human cancer development [39, 40].

However, data on ERα expression in the canine prostate are inconsistent. Canine prostatic carcinoma can arise from acinar, ductal or urethral epithelial cells, with mixed morphology within the same primary tumor a frequent observation [34, 41]. In men, urothelial tumors are histomorphologically distinguished from primary glandular carcinomas, however in dogs this can be difficult as tumors are often poorly differentiated at the time of diagnosis [28, 34, 41]. Some studies have reported absent or duct‐limited ERα expression in normal canine epithelium [42], whilst others have demonstrated high ERα expression in glandular cells [43, 44] and ductal cells [45]. In the studies that observed ERα positivity in non‐malignant canine epithelial cells, all found expression to be significantly reduced with malignancy [43, 45].

Stromal ERα expression in the canine prostate is more consistently observed, particularly in smooth muscle cells [42, 43, 45, 46]. In contrast with human tissue, ERα expression in prepubertal dogs has been reported to be limited to the stroma, with stromal expression highest in this age group when compared with all other life stages [42].

Castration is routinely carried out in dogs for behavioural and reproductive control in many western countries [47, 48]. Although some studies have reported higher PCa incidence post‐castration [32, 49, 50, 51], others have found no significantly increased PCa risk in castrated dogs compared with those that remain sexually intact [31, 34, 52], or between dogs castrated pre‐ or postpubertally [50]. Notwithstanding some methodological inconsistencies between studies, there is some consensus that neutered dogs do have an increased risk of developing PCa of urothelial origin, rather than general PCa [49, 53, 54]. Given the prevalence of PCa in both castrated and entire dogs, canine PCa appears to be less androgen‐dependent than its human counterpart.

Interestingly, this may parallel emerging clinical scenarios in transgender women. Although prepubertal castration in men was historically thought to eliminate PCa risk [55], no longitudinal data confirm this in the modern era. Transgender women undergoing male‐to‐female transition (via orchiectomy and estrogen therapy) represent a markedly growing demographic, with gender reassignment treatments tripling between 2016 and 2019 in the USA alone [55, 56]. Recent estimates suggest ~1.6 million individuals in the USA identify as transgender, of which ~42% are under the age of 25 [47]. The prostate is not removed during male‐to‐female gender reassignment surgery due to the risk of significant side effects, however studies have demonstrated that biological activity persists in the prostate even after orchiectomy [48, 55]. Despite the low incidence of PCa ( ~ 0.04%) in transgender women, reported cases tend to be aggressive and, by definition, castration‐resistant [55], a disease pattern that mirrors canine PCa.

Due to variable ERα expression and conflicting data across the commonly used *in vitro* PCa models [24, 57, 58], it has been suggested that traditional cell lines are inappropriate for studying the role of estrogen signaling in PCa, and that alternative models are required [58]. This lack of reliable ERα expression in common *in vitro* PCa cell lines [58] and significant differences between human prostate anatomy and histology and that of rodents, our most commonly used animal PCa model to date [59, 60], highlight a need for a more compatible animal model. Given the marked increase in the number of transgender women that will be progressing into higher risk age groups for cancer in the coming years, there is an urgent need for greater understanding of estrogen‐driven prostate carcinogenesis. The current study therefore investigated the value of the canine model in furthering our understanding of the role of ERα in human PCa.

## 2 Materials and Methods

### 2.1 Samples

This study was ethically reviewed and approved by the local ethics committee of the University of Nottingham School of Veterinary Medicine and Science (1669 160208, 1861 161006 and 3483 211102) and the NUH NHS Trust Biobank Access Committee (ACP0000184). The Human Tissue Act and Helsinki Declaration of Human Rights were strictly observed, and the General Data Protection Regulation (GDPR) was applied.

A human prostate tissue microarray (TMA) was constructed by a certified pathologist (MST) using prostatectomy specimens from a patient cohort diagnosed with PCa (*n* = 104) between 2003 and 2007 at the Nottingham University Hospitals (NUH) NHS Trust (Table 1). The TMA comprised 160 formalin‐fixed paraffin‐embedded (FFPE) 0.6 mm tissue cores in a single block. Each of the 104 Nottingham patients was represented by one primary prostate adenocarcinoma specimen, with the non‐malignant specimens taken from 56 members of the same patient cohort. Due to missing or uninterpretable cores, seven adenocarcinoma and seven non‐malignant specimens were not available for analysis.

**Table 1:** Summary of patient demographics and clinicopathological parameters analysed in the Nottingham prostate TMA patient cohort (pTNM= patient tumor, node, metastasis; PSA= prostate specific antigen).

Parameter		Number of patients
Age (years)	≤ 60	44
	≥ 61	60
Ethnicity	White	94
	Mixed/Black Caribbean	2
	Any other	2
	N/A	6
Gleason score	3 + 3	12
	3 + 4	46
	4 + 3	22
	8 or 9	23
	N/A	1
pTNM	T1&T2	66
	T3	33
	N/A	5
Perineural invasion	No	32
	Yes	72
Positive surgical margins	No	55
	Yes	47
	N/A	2
Extraprostatic extension	No	66
	Yes	36
	N/A	2
Preoperative PSA levels (ng/ml)	≤ 10	55
	≥ 11	46
	N/A	3
Biochemical recurrence	No	59
	Yes	33
	Never tumor free	12
Time to biochemical recurrence (months)	≤ 23	15
	≥ 24	18

FFPE prostate tissue sections from a cohort of 61 male pet dogs [*n* = 34 carcinoma, *n* = 17 benign gland, *n* = 10 premature gland] were also included following assessment by two board‐certified veterinary pathologists (Table 2). The canine tissue specimens were obtained between 2009 and 2018 from both the University of Nottingham Pathology Service and Bridge Pathology in Bristol, UK. Tumor tissues were obtained from excisional or incisional biopsies. Benign prostatic tissues were collected at necropsy from dogs that had died of non prostate‐related causes. Benign samples comprised both normal non‐malignant tissue and benign prostatic hyperplasia (BPH), as mixed forms were common. Atrophy in benign glands was diffuse and present in all castrated dogs (n = 4), yet mild and focal when present in 10 of 13 entire dogs. Inflammation, if present, was mild, characterized by a predominantly lymphoplasmacytic stromal infiltrate, with associated intralesional glandular atrophy. Of the 17 adult dogs with prostate tissue assessed as normal or benign, four were surgically castrated, whereas 21 of the 34 dogs with PCa were castrated. Castration status was unknown for six dogs. Premature dogs, which were defined as those less than 6 months of age with a prostate gland histologically exhibiting non‐secretory epithelium, were all entire. Tissue from all ten premature dogs was assessed as non‐malignant.

**Table 2:** Overview of the studied canine cases (*n* = 17 benign; *n* = 10 premature; *n* = 34 tumor).

Case	Group	Prostate tissue histopathology^a^						Neutered^b^	Breed	Age (years)
		Normal	Premature	BPH	Atrophy	Tumor	Inflammation			
1	Benign	0	0	1	1	0	0	0	English Bulldog	7
2	Benign	0	0	1	1	0	0	0	Giant Schnauzer	10
3	Benign	1	0	0	0	0	0	0	Staffordshire Bull Terrier	11
4	Benign	0	0	0	1	0	0	1	Chihuahua	8
5	Benign	0	0	1	1	0	1	0	West Highland White Terrier	11
6	Benign	1	0	0	1	0	1	0	English Springer Spaniel	3
7	Benign	1	0	0	1	0	1	0	Dogue de Bordeaux	4
8	Benign	1	0	1	1	0	1	0	Boxer	7
9	Benign	0	0	0	1	0	0	1	Greyhound	8
10	Benign	1	0	0	1	0	1	0	Greyhound	3
11	Benign	0	0	0	1	0	0	1	French Bulldog	3
12	Benign	1	0	0	1	0	0	0	Miniature Australian Shepherd	1
13	Benign	1	0	0	1	0	0	0	Malinois	7
14	Benign	1	0	0	0	0	0	0	Miniature Poodle	0 (10 months)
15	Benign	0	0	0	1	0	0	1	Border Terrier	5
16	Benign	0	0	1	0	0	0	0	GSD X Rottweiler	9
17	Benign	1	0	0	1	0	1	0	Australian Shepherd	0 (8 months)
TOTAL	Benign (*n* = 17)	9	0	5	14	0	6	5		
18	Premature	0	1	0	0	0	0	0	Leonberger	0 (2 weeks)
19	Premature	0	1	0	0	0	0	0	Boxer	0 (6 weeks)
20	Premature	0	1	0	0	0	0	0	Labrador	0 (3 months)
21	Premature	0	1	0	0	0	0	0	Cross breed	0 (4 months)
22	Premature	0	1	0	0	0	0	0	Cavalier King Charles Spaniel	0 (8 weeks)
23	Premature	0	1	0	0	0	0	0	Leonberger	0 (2 weeks)
24	Premature	0	1	0	0	0	0	0	Leonberger	0 (2 weeks)
25	Premature	0	1	0	0	0	0	0	Pointer	0 (5 months)
26	Premature	0	1	0	0	0	0	0	Cavalier King Charles Spaniel	0 (5 months)
27	Premature	0	1	0	0	0	0	0	French Bulldog	0 (3 months)
TOTAL	Premature (*n* = 10)	0	10	0	0	0	0	0		
28	Tumor_Ca	0	0	0	0	1	0	1	Fox Terrier	6
29	Tumor_Ca	0	0	0	0	1	0	1	Samoyed	10
30	Tumor_Ca	0	0	0	0	1	0	NA	Labrador Retriever	10
31	Tumor_Ca	0	0	0	0	1	0	1	Labrador Retriever	8
32	Tumor_Ca	0	0	0	0	1	0	1	Jack Russell Terrier	NA
33	Tumor_Ca	0	0	0	0	1	0	1	Labrador Retriever	11
34	Tumor_Ca	0	0	0	0	1	0	NA	Cross breed	10
35	Tumor_Ca	0	0	0	0	1	0	1	Airedale Terrier	9
36	Tumor_Ca	0	0	0	0	1	0	0	Flat‐coated Retriever	9
37	Tumor_Ca	0	0	0	0	1	0	1	Labrador Retriever	9
38	Tumor_Ca	0	0	0	0	1	0	0	Labrador Retriever	10
39	Tumor_PC	0	0	0	0	1	0	NA	Giant Schnauzer	NA
40	Tumor_PC	0	0	0	0	1	0	1	Labrador Retriever	10
41	Tumor_PC	0	0	0	0	1	0	0	Rottweiler	7
42	Tumor_PC	0	0	0	1	1	0	1	Jack Russell Terrier	15
43	Tumor_PC	0	0	0	0	1	0	0	Labrador Retriever	11
44	Tumor_PC	0	0	0	0	1	1	0	Border Collie	10
45	Tumor_PC	0	0	0	0	1	0	NA	Boxer	10
46	Tumor_PC	0	0	0	0	1	0	0	Rottweiler	5
47	Tumor_PC	0	0	0	0	1	0	0	Springer Spaniel	9
48	Tumor_UC	0	0	0	0	1	0	1	Yorkshire Terrier	7
49	Tumor_UC	0	0	0	0	1	0	1	Labrador Retriever	9
50	Tumor_UC	0	0	0	0	1	0	1	Labrador Retriever	9
51	Tumor_UC	0	0	0	0	1	0	1	Cross breed	13
52	Tumor_UC	0	0	0	0	1	0	1	Labrador Retriever	12
53	Tumor_UC	0	0	0	0	1	0	1	Yorkshire Terrier	12
54	Tumor_UC	0	0	0	0	1	0	1	Miniature Schnauzer	11
55	Tumor_UC	0	0	0	0	1	0	1	Corgi	11
56	Tumor_UC	0	0	0	0	1	0	1	Cross breed	9
57	Tumor_UC	0	0	0	0	1	0	NA	Weimaraner	10
58	Tumor_UC	0	0	0	0	1	0	NA	Cocker Spaniel	9
59	Tumor_UC	0	0	0	0	1	0	1	Staffordshire Bull Terrier	10
60	Tumor_UC	0	0	0	0	1	0	1	Cross breed	10
61	Tumor_UC	0	0	0	0	1	0	1	Hungarian Vizsla	11
TOTAL	Tumor (*n* = 34)	0	0	0	1	34	1	21		

Abbreviations: BPH, benign prostate hyperplasia; Ca, carcinoma (not further defined); GSD, German Shepherd Dog; NA, not applicable/not available; PC, prostate carcinoma; UC, urothelial carcinoma.

^a^ as present on the studied prostate tissue section; 1: present; 0: absent

^b^ 1: yes; 0: no; NA: unknown

### 2.2 Histology

Histological assessment of hematoxylin and eosin (HE) stained sections of human tissue was carried out by a certified pathologist (MST) at City Hospital, Nottingham and classified as either prostate adenocarcinoma or non‐malignant tissue. Representative cores were subsequently selected for inclusion in the TMA.

HE stained full tissue sections of canine prostate were assessed by two board‐certified veterinary pathologists (LA, SdB). All prostate sections were assessed for the presence of tumor, benign prostatic hyperplasia (BPH), glandular atrophy and inflammation. Canine prostate tumors (*n* = 34) were classified based on histomorphology as either prostatic adenocarcinoma (PC) (*n* = 9), or prostatic urothelial carcinoma (UC) (*n* = 14). Mixed urothelial and glandular or not further classifiable phenotypes were classified as prostatic carcinoma (Ca) (*n* = 9) as previously described [61]. Canine PC samples were considered to be most representative of human PCa given their identical cell of origin and similar histopathological appearance.

### 2.3 Immunohistochemistry

Immunohistochemistry (IHC) of the human TMA was performed using the Novolink Max Polymer Detection System (Leica Biosystems, UK) as previously described [62]. Staining of the 4 µm TMA section was carried out using a primary monoclonal anti‐human ERα antibody (1:20, M3634 clone SP1, Dako, Denmark), incubated overnight at 4°C. Following staining, the slide was scanned at high resolution using the Pannoramic 250 Flash III (3D Histech, Hungary).

IHC of the canine tissue sections was performed using the BOND‐III Fully Automated IHC and ISH Staining System (Leica Biosystems, Switzerland). Two to three µm paraffin‐embedded full‐face tissue sections were stained as previously described [63]. For pretreatment, endogenous peroxidase activity was blocked in a solution of H_2_O_2_ with epitope retrieval buffer type 2 (Tris‐EDTA, pH 9 (Leica Biosystems, Switzerland)) subsequently used. The tissue was then incubated for 15 min at room temperature with the primary monoclonal anti‐human ERα antibody (1:50, M3643 clone EP1, Dako, Denmark). This specific antibody has been previously validated for diagnostic use in the same laboratory and has confirmed cross‐reactivity and high specificity in canine tissue [63]. Staining of known positive (canine mammary gland) and negative (no primary antibody) controls was carried out alongside each series.

Following staining, the slide was scanned at 40x resolution using the S360 Nanozoomer (Hamamatsu Photonics, Japan). All canine whole slide images (WSI) and the human TMA image were subsequently assessed by a board‐certified veterinary pathologist (SdB) to ensure sufficient tissue, staining and scanning quality prior to digital analysis.

### 2.4 Digital ERα Expression Analysis

Digital assessment of IHC staining was carried out using commercial artificial intelligence‐driven precision pathology software. Visiopharm (version 2024.07, Horsholm, Denmark) was utilized to create a fully quantitative and spatial deep‐learning (DL) analysis of nuclear ERα expression. The following workflow was applied: (1) Tissue detection and segmentation into gland and stroma which were defined as regions of interest (ROI) (DL classification (U‐Net); 80 K iterations); (2) Revision of ROIs with manual corrections where needed and manual assignment to specific epithelial subcategories (benign, premature, atrophy, tumor, urethra) where applicable by two board‐certified veterinary pathologists; (3) Detection and labeling of individual cell nuclei (DL classification (U‐Net); 100 K iterations) with classification as negative, weak, moderate or strongly ERα positive based on the HDAB – DAB feature mean pixel values per nucleus (change label by intensity post processing step considering 25–75% of the object; negative > 170; weak 120–170; moderate 70–120; strong < 70 mean HDAB‐DAB pixel values); (4) Generation of output: Assessed epithelial (with subcategories benign, premature, atrophy, tumor and urethra) and stromal tissue area (mm^2^); total counts of negative, weak, moderate and strongly ERα positive nuclei per ROI; calculation of H‐scores (defined as (1 × percentage of weakly stained nuclei) + (2 × percentage of moderately stained nuclei) + (3 × percentage of strongly stained nuclei), ranging from 0 to 300) for each ROI; (5) Generation of ERα expression heatmaps, based on the positive nuclear counts, for visualization of the spatial marker expression.

### 2.5 Statistical Analysis

Statistics were performed using NCSS 2024 Statistical Software (NCSS LLC. Kaysville, Utah, USA, ncss. com/software/ncss). Normality of the data (*H*‐scores) was assessed using the Shapiro–Wilk test which confirmed that the data did not follow a normal distribution. Comparisons of *H*‐scores between groups were performed using the nonparametric Mann–Whitney *U* test (Wilcoxon rank‐sum test). The following groups were compared: species (canine vs. human), tissue type (premature, benign, tumor with subcategories prostate carcinoma, carcinoma not further defined and urothelial carcinoma) and tissue compartment (gland vs. stroma). Levene's test for equality of variances was used to evaluate the homogeneity of variances across groups. *p* < 0.05 was considered significant.

## 3 Results

**Figure 1:**
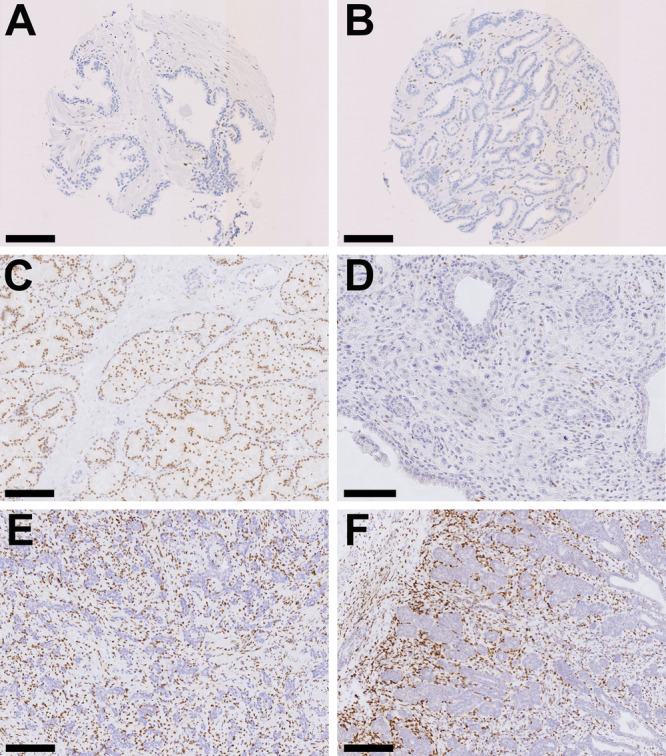
Microphotographs of human and canine prostate tissue. Estrogen receptor alpha (ERα) immunohistochemistry. Size bar indicates 150 µm in A, B, C, E and F and 80 µm in D. (A) Normal human prostate with negative glandular epithelium and few positive stained cells in the stroma. (B) Human prostate carcinoma. Positive cells are observed in the stroma, whereas tumor cells remain negative. (C) Normal canine prostate. Diffuse positive glandular nuclear staining is evident with low numbers of positive stromal cells. (D) Canine prostate carcinoma. Identical to human carcinoma, tumor cells are negative and rare positive stromal cells are observed. (E) Canine prostate atrophy. Diffuse stromal staining is present with lack of ERα expression in the atrophic glands. (F) Canine premature prostate. Diffuse strong stromal staining which is more pronounced towards the periphery of the prostate (towards left). The non‐secretory epithelium lacks ERα expression. [Color figure can be viewed at wileyonlinelibrary.com]

**Figure 2:**
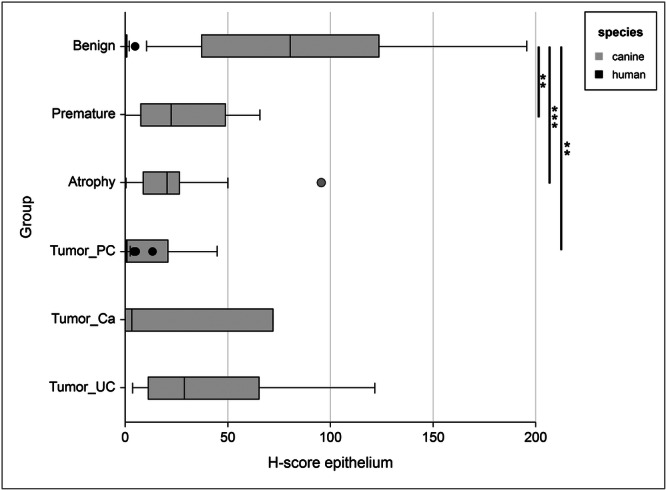
Box plot illustrating the H‐scores of ERα expression in the prostate epithelium of men and dogs, comparing species and tissue categories. Statistical significance refers to comparisons of different canine tissue categories; *p* < 0.05 (*), *p* < 0.01 (**), *p* < 0.001 (***). Abbreviations: Ca, carcinoma (not further defined); PC, prostate adenocarcinoma; UC, urothelial carcinoma.

Prostate tissue from 104 men (Table 1) and 61 dogs (Table 2) was examined for ERα expression and compared (Figures 1 and 2).

Overall, ERα staining showed distinct species and tissue‐specific patterns. In human prostate tissue ERα positive cells were rare or absent in both benign and malignant glandular epithelium, indicating negligible ERα expression. In the prostatic stroma, only occasional positive cells were observed, primarily corresponding to smooth muscle fibers. In contrast, canine tissues exhibited diffuse nuclear ERα positivity in benign glands, whereas lower and more heterogeneous expression was observed in tumor tissue. Atrophic and immature glands lacked detectable ERα expression.

The stromal compartment of both benign and malignant canine prostate tissue displayed a distribution pattern similar to that in humans, with scattered ERα‐positive cells, typically representing smooth muscle cells. However, stromal ERα expression was markedly increased in premature canine prostates.

Expression of ERα in the glandular epithelium of the human prostate samples was very low to absent across both non‐malignant and prostatic adenocarcinoma tissue (Figure 1A and B). In non‐malignant prostate specimens (*n* = 49) the mean H‐score was 0.6 (median 0.3; SD 0.9; SEM 0.1; range 0–5), with no significant difference (*p* = 0.33) in ERα expression observed in the adenocarcinoma specimens (*n* = 97) (mean *H*‐score 0.9; median 0.2; SD 1.7; SEM 0.2; range 0–13). No significant correlation was observed between glandular ERα expression and any of the clinicopathological parameters detailed in Table 1.

Significantly higher ERα expression was found in the glandular epithelium of benign prostatic tissue from mature dogs (*n* = 17; Figure 1C; mean H‐score of 85 (median 80; SD 55; SEM 14; range 10.3–195.7)) than in non‐malignant human specimens (*n* = 49; *p* < 0.0001; Figure 1A). Similarly, ERα expression was also significantly higher in canine tumor tissue (*n* = 34, Figure 1D) than in human (*n* = 97; *p* = < 0.0001). Table 3 details all *H*‐scores from both human and canine cohorts.

**Table 3:** Estrogen receptor alpha expression in the studied human and canine prostate tissue. Indicated are H‐scores of the immunohistochemical nuclear ERα staining.

H‐score; mean (standard deviation)	Prostate tissue					
Epithelium	Benign	Tumor (PC)	Premature	Atrophy	Ca	UC
	(H *n* = 49)	(H *n* = 97)				
	(C *n* = 17)	(C *n* = 9)	(C *n* = 10)	(C *n* = 24)	(C *n* = 11)	(C *n* = 14)
human	0.6 (0.9)	0.9 (1.7)	NA	NA	NA	NA
*p* (tissue)	‐‐‐	0.332	‐‐‐	‐‐‐	‐‐‐	‐‐‐
canine	85 (55)	10 (16)	27 (24)	24 (22)	51 (81)	48 (50)
*p* (tissue)	‐‐‐	**0.006**	**0.007**	**< 0.0001**	0.198	0.063
*p* (species)	**< 0.0001**	**< 0.0001**	NA	NA	NA	NA
Stroma	Benign	Tumor (PC)	Premature	Atrophy	Ca	UC
human	6 (8)	6 (8)	NA	NA	NA	NA
*p* (tissue)	‐‐‐	0.786	‐‐‐	‐‐‐	‐‐‐	‐‐‐
canine	19 (17)	26 (42)	74 (45)	43 (37)	27 (29)	44 (30)
*p* (tissue)	‐‐‐	0.0043	**0.0036**	0.019	0.393	**0.009**
*p* (species)	**< 0.0001**	**< 0.0001**	NA	NA	NA	NA
Ratio epithelium:stroma	Benign	Tumor (PC)	Premature	Atrophy	Ca	UC
human	0.1 (0.2)	0.1 (0.1)	NA	NA	NA	NA
*p* (tissue)	‐‐‐	0.256	‐‐‐	‐‐‐	‐‐‐	‐‐‐
canine	7 (7)	0.6 (1.2)	0.4 (0.2)	1.1 (1.7)	1 (2)	2 (3)
*p* (tissue)	‐‐‐	**0.011**	**0.005**	**0.0002**	**0.021**	**0.011**
p (species)	**< 0.0001**	**< 0.0001**	NA	NA	NA	NA

*Note:* Statistically significant *p* values are in bold.

Abbreviations: Ca, carcinoma (not further defined); C, canine; H, human; NA, not applicable/available; PC, prostate carcinoma; UC, urothelial carcinoma

The highest level of ERα expression was observed in canine non‐malignant epithelium that sat adjacent to tumor tissue (mean *H*‐score 145; median 173; SD 69; SEM 40; range 66–196), however this was only available for evaluation in three specimens. Epithelial ERα expression was significantly reduced in canine PC specimens (*n* = 9; *p* < 0.0001) compared with mature canine benign prostate tissue.

In dogs, significantly lower epithelial ERα expression was also observed in atrophic prostatic tissue (*n* = 24; *p* = < 0.0001) compared with mature benign epithelium, and was consistent across both atrophic benign (*n* = 13; of which *n* = 4 diffuse atrophy, *n* = 9 focal or multifocal atrophy) and atrophic tumor (*n* = 11; of which *n* = 7 UC, *n* = 3 PC, *n* = 1 Ca; *p* = < 0.0001) canine specimens (Figure 1E). Significantly lower epithelial ERα expression was also observed in tissue from premature dogs (*n* = 10; *p* = 0.007; Figure 1F) than in mature benign epithelium. In canine prostate tissue sections with normal or hyperplastic glands admixed with atrophic glands, ERα was expressed in active (secretory) prostatic glands, whereas expression was low to absent in adjacent atrophic gland profiles (Supplementary Figure 1).

Loss of epithelial ERα expression with neoplastic transformation was observed in dogs across all tumor types, but was most evident in the PC tumor specimens (*n* = 9; *p* = 0.006). No significant difference in epithelial ERα expression was observed between tumor (all subtypes combined), atrophic or premature samples, or between Ca and either PC or UC tumor types. However, ERα expression was significantly reduced in the PC specimens compared with UC (*p* = 0.04). Neutering status did not significantly affect epithelial ERα expression between PC, UC or Ca tumor types.

A heterogeneous pattern of ERα expression was observed in malignant epithelial cells in 17/34 canine tumor specimens (2 PC, 12 UC, 3 Ca). In five cases, the heterogeneity of ERα expression was associated with the histomorphology of the tumor (Supplementary Figure 2). Specifically, in one Ca specimen, ERα was not expressed in an area of highly pleomorphic epithelial cells with an invasive appearance, although expression was observed in more compact urothelial regions (Supplementary Figure 2A). Similarly, in a second Ca sample, highly invasive small glands and nests were negative for ERα expression, whereas areas with a more confined glandular cribriform growth were positive (Supplementary Figure 2B). In a UC specimen, ERα expression was not observed in cribriform areas, although it was present in regions with more vacuolated growth of malignant cells (Supplementary Figure 2C). In a second UC specimen, larger, more pleomorphic and invasive tumor regions were negative for ERα expression, whereas small to medium‐sized solid‐cribriform glands were positive (Supplementary Figure 2D‐F). In a third UC case, cribriform areas of tumor remained negative for ERα expression, whilst solid tumor regions were positive.

**Figure 3:**
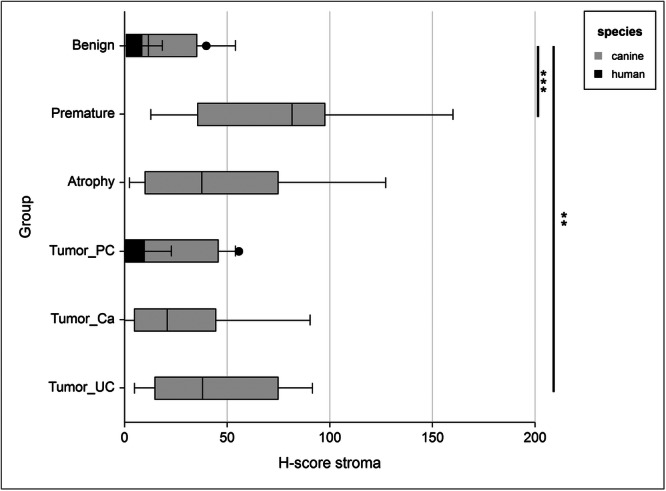
Box plot illustrating the H‐scores of ERα expression in the prostate stroma of men and dogs, comparing species and tissue categories. Statistical significance refers to comparisons of different canine tissue categories; *p* < 0.05 (*), *p* < 0.01 (**), *p* < 0.001 (***). Abbreviations: Ca, carcinoma (not further defined); PC, prostate adenocarcinoma; UC, urothelial carcinoma.

Stromal expression of ERα in the human prostate samples was low, although more variable than in the epithelium (Figures 1 and 3). In non‐malignant prostate specimens (*n* = 49) the mean H‐score was 6 (median 4; SD 8; SEM 1; range 0–40; Figure 1A), with no significant difference in ERα expression observed in the adenocarcinoma specimens (*n* = 104; mean H‐score 6; median 3; SD 8; SEM 1; range 0–56; Figure 1B). No significant correlations were observed between stromal ERα expression and any of the clinicopathological parameters evaluated. The ratio of epithelial:stromal ERα expression was assessed, however no significant difference was observed between non‐malignant (mean 0.1; median 0.1; SD 0.2; SEM 0.03, range 0–1.3) and tumor (mean 0.1; median 0; SD 0.1; SEM 0.01, range 0–0.6) tissue (Table 3).

In dogs, stromal ERα expression in mature benign tissue was significantly higher overall than in human non‐malignant stroma (*p* = < 0.0001), but was still generally low (Figure 1C), with a mean H‐score of 19 (median 12; SD 17; SEM 4; range 2–54).

In contrast to the canine epithelial findings, higher stromal ERα expression was observed in all canine tumor specimens (Figure 1D) compared with mature canine benign tissue (*p* = 0.0446), although this observation did not reach statistical significance with the PC specimens alone. The highest stromal expression of ERα was seen in benign tissue from premature dogs (mean H‐score 74; median 82; SD 45; SEM 14; range 13‐160), and was significantly higher than that observed in canine mature benign (*p* = 0.0036) and malignant tissue (Figure 1E), both for all tumor subtypes combined (*p* = 0.0043) and PC specimens alone (*p* = 0.0286). No significant difference in stromal ERα expression was observed between Ca and either PC or UC tumor types.

When considering the spatial distribution of ERα in canine stroma, expression in non‐malignant tissue was observed primarily in smooth muscle cells. In premature canine prostate tissue, stromal ERα was characterized by a specific distribution pattern which was similar in all examined cases. The highest levels of ERα expression were observed in the most peripheral gland regions and around the ductus deferens (Figure 1F). Moderate ERα expression was seen in the periurethral areas, with overall lower ERα expression in the remaining prostate regions (Supplementary Figure 3). Stromal ERα expression was observed to reduce with increasing age and, therefore, advancing prostate development (Figure 3). In the stroma of atrophic non‐malignant prostate tissue ERα was primarily expressed in the smooth muscle, whilst the epithelium remained largely negative (Figure 1F). Malignant prostate tissue revealed no specific spatial stromal ERα expression patterns except for the high affinity for smooth muscle, as observed in benign tissue.

The ratio of epithelial:stromal ERα expression in canine prostates was assessed and found to be significantly lower in tumor tissue of all subtypes (mean 1.3; median 0.3; SD 2; SEM 0.4, range 0–8) compared with mature benign prostatic tissue (mean 7; median 5; SD 7; SEM 2, range 2–30; *p* = 0.0045). When only PC cases were evaluated, the epithelial:stromal ratio was even lower (mean 0.6; median 0.2; SD 1.2; SEM 0.4, range 0–4; *p* = 0.011; Table 3). No significant differences in the ratios of epithelial:stromal ERα expression were found between premature (mean 0.4; median 0.3; SD 0.2; SEM 0.1), atrophic (mean 1.1; median 0.5; SD 1.7; SEM 0.3) or malignant prostatic tissue.

## 4 Discussion

ERα expression was examined in prostatic tissue samples from a cohort of 104 PCa patients. In the glandular epithelium, expression of ERα was essentially negative across both non‐malignant and malignant specimens, with mean *H*‐scores of 0.6 and 0.9, respectively. Stromal ERα expression was also low (mean *H*‐score 5), although more variable than in the epithelium. These findings accord with many previous studies [11, 23, 24, 25, 26, 64].

The current study found no significant differences in ERα expression in either the glandular epithelium or the stroma between human non‐malignant and PCa specimens, or across ranges of age or Gleason grade. A large multicenter study comprising over 500 PCa patients similarly found no correlation between epithelial ERα expression and any of the clinicopathological parameters it examined, although it did report a significant positive correlation between stromal ERα expression and delayed time to clinical failure and PCa death [64]. Whilst a previous smaller study conversely reported a significant positive correlation between ERα expression and PCa progression, it should be noted that no distinction between epithelial and stromal expression was made, staining intensity was not evaluated as part of their semi‐quantitative IHC scoring system, and the patient cohort comprised only 28 men [23].

Significantly higher ERα expression was observed in the benign glandular epithelium of mature dogs than men. Similar findings have previously been described, with high levels of ERα expression reported in canine non‐malignant glandular epithelial cells [43, 44, 45]. Likewise, the current study found that ERα expression was significantly higher in neoplastic canine epithelium than human. To the best of the authors’ knowledge, this is the first time a direct comparison between canine and human ERα expression in the prostate has been reported.

ERα expression was observed to significantly reduce with neoplastic transformation in canine tumor samples compared with mature canine benign epithelium. The current study had access to tissue from dogs across a wide age range, thus enabling examination of ERα expression in premature, prepubertal dogs, through to older dogs with prostatic atrophy. Only low level ERα expression was observed in non‐malignant glandular epithelium from premature dogs. This was significantly lower than the ERα expression seen in the epithelium of mature dogs. The authors are only aware of one study that has examined ERα expression in normal glandular cells from juvenile dogs, and it found them to be ERα negative [65].

The current study also found similar low levels of ERα expression in atrophic canine non‐malignant and malignant prostatic tissue, both significantly lower than that observed in mature non‐atrophic tissue. To the best of the authors’ knowledge, the current study is the first time that the level of ERα expression in atrophic canine prostatic tissue has been reported. It is widely accepted that surgical and chemical castration results in significant prostatic atrophy in men [66, 67, 68]. The resulting reduction of circulating androgen hormones results in loss of androgen signaling, inactivity of the AR, and the dedifferentiation of luminal epithelial cells in the prostate [69]. The impact of prostatic atrophy on ERα expression is very poorly understood in both men and dogs. The canine findings reported in the current study indicate further work to elucidate the role of ERα in castrate‐resistant PCa in men.

As human stroma was generally ERα negative, stromal ERα expression in dogs was significantly higher in both non‐malignant and tumor samples when compared with their human counterparts.

In dogs, ERα was expressed at a generally low level in the stroma of benign prostatic tissue, in accordance with earlier studies [44, 45, 46, 65]. Whilst a trend of higher stromal ERα expression was observed in canine neoplastic tissue compared with benign, this did not reach statistical significance. Likewise, no significant difference in stromal ERα expression was observed between benign and malignant tissue from mature dogs, or between the PC, UC or Ca tumor types. Other studies have reported varying levels of stromal ERα expression in non‐malignant and neoplastic canine prostatic tissue [43, 45, 46, 65]. However, low patient numbers and a variety of methods of assessing and quantifying ERα expression make interpretation and comparison of their findings challenging.

Significantly higher ERα expression was observed in the prostatic stroma of premature dogs compared to mature benign or malignant specimens. Stromal ERα was primarily expressed in smooth muscle cells, both in non‐malignant and neoplastic canine tissue, as previously reported [45, 70]. A specific distribution pattern of stromal ERα expression was observed in the premature specimens, with the highest expression around the ductus deferens and periphery that then decreased with age. Similar high ERα expression in ductal cells has also been reported in mature canine specimens [42, 45, 65], although the authors are not aware of any other published observations of ERα expression distribution in premature dogs. To the best of the authors’ knowledge, the current study comprises the greatest number of dogs in a single study for the examination of prostatic stromal ERα expression to date.

Whilst it should be noted that the distribution of neutered dogs between PC (2/7 neutered, 2 unknown neuter status; total 9) and UC (12/12 neutered) cases was unequal, the current study found no significant correlation between neutering status and ERα expression between any of the PC, UC or Ca tumor types. Whilst the authors are not aware of any other studies examining the effect of castration on ERα expression in canine PCa, one study comparing dogs with normal prostates to those with BPH similarly found that castration had no significant effect on prostatic ERα expression in either group [46].

Whilst a TMA was used to analyze the human specimens in this study, full‐face tissue sections were available from the canine patients, allowing for detailed evaluation of the spatial distribution of ERα expression across the prostate. This histomorphological assessment found that areas with lower ERα expression appeared to be indicative of malignancy and disease progression. As the human TMA specimens generally lacked ERα expression in the current study, it would be helpful in future work to be able to assess the full‐face tissue sections from our human patient cohort for evidence of a similar trend of spatial distribution. This availability of abundant tissue for evaluation in veterinary pathology is another key advantage in using the canine PCa model, as tissue availability is often much more limited with human specimens.

Dogs provide a valuable opportunity to further our understanding of the role of ERα in human PCa. Not only are they the only mammalian species to spontaneously develop PCa with any regularity, there are also a number of similarities in the pathogenesis of CRPC in men and PCa in dogs. Like human CRPC, canine PCa is androgen‐independent and shares the same histomorphological appearance and metastatic tropisms. Additionally, we and others have demonstrated that ERα is expressed in both the human and canine prostatic stroma.

Taken together, these findings support the utility of the canine prostate as a valuable comparative model to further elucidate the role of ERα in the pathogenesis and progression of human CRPC.

## 5 Conclusions

This study demonstrates that malignant glandular epithelium lacks ERα expression in both dogs and men, with neoplastic transformation in the canine prostate accompanied by a shift in ERα expression from the epithelium to the stroma, highlighting a dynamic and context‐dependent role for ERα within the tumor microenvironment. Unlike in men, benign canine glands show diffuse ERα expression, whereas premature and atrophic glands display very low ERα levels. The observed differences in ERα expression across prostate tissue types in the dog —premature, normal, atrophic, and tumor—warrant further investigation to provide a clearer understanding of the role of ERα in PCa progression and therapeutic response, particularly in advanced castration‐resistant disease. These insights are especially pertinent in light of the growing number of young transgender women undergoing orchiectomy and exogenous estrogen treatment. As this population progresses into higher risk age categories, understanding the role of ERα in CRPC biology will be crucial for developing effective PCa screening and management strategies.

## Conflicts of Interest

The authors declare no conflicts of interest.

## Supporting information


**Supporting Figure S1:** Heatmap of ERα expression in a cross section of canine prostate tissue comprising secretory (black outline) and atrophic (red outline) gland profiles.

**Supporting Figure S2:** Microphotographs of canine prostate tumors with heterogenous ERα expression.

**Supporting Figure S3:** Microphotograph of a premature canine prostate of a 2‐week old puppy, case 18.

**Supporting Figure S4:** Scatter plot illustrating the correlation between age and stromal ERα expression (*H*‐score) in canine premature prostate tissue.

## Data Availability

The data that support the findings of this study are available from the corresponding author upon reasonable request.
