# Supplementary figures and images for: Comparative Digital Estrogen Receptor Alpha (ERα) Expression Analysis in Benign and Malignant Prostate Tissue of Men and Dogs
Source: Prostate. 2025 Dec 23;86(5):568–81. doi: 10.1002/pros.70111 (PMC12935393; doi:10.1002/pros.70111)

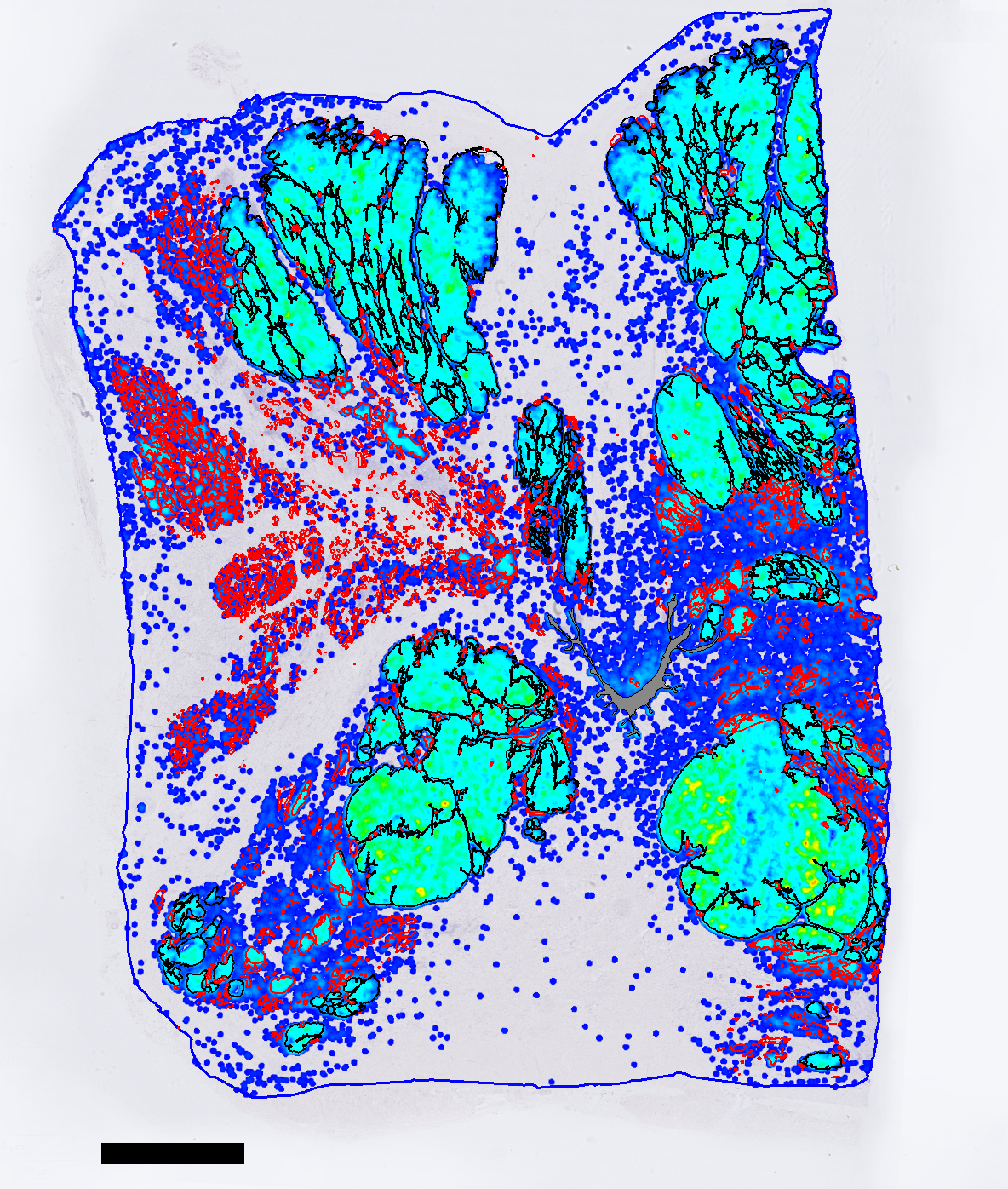

Supplement: Supplementary file 1 — Supporting Figure S1: Heatmap of ERα expression in a cross section of canine prostate tissue comprising secretory (black outline) and atrophic (red outline) gland profiles. [file PROS-86-568-s004.tif]

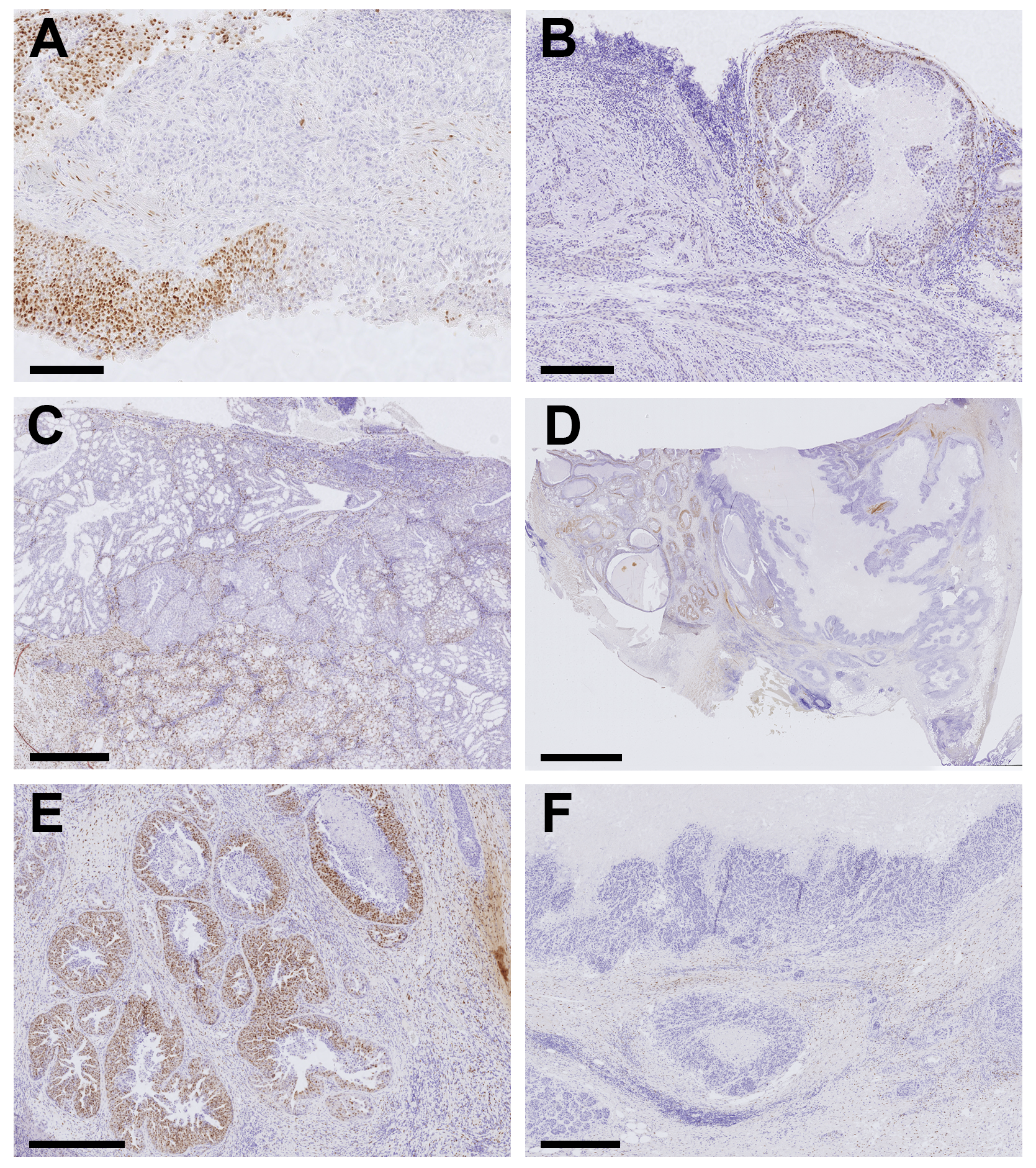

Supplement: Supplementary file 2 — Supporting Figure S2: Microphotographs of canine prostate tumors with heterogenous ERα expression. [file PROS-86-568-s003.tif]

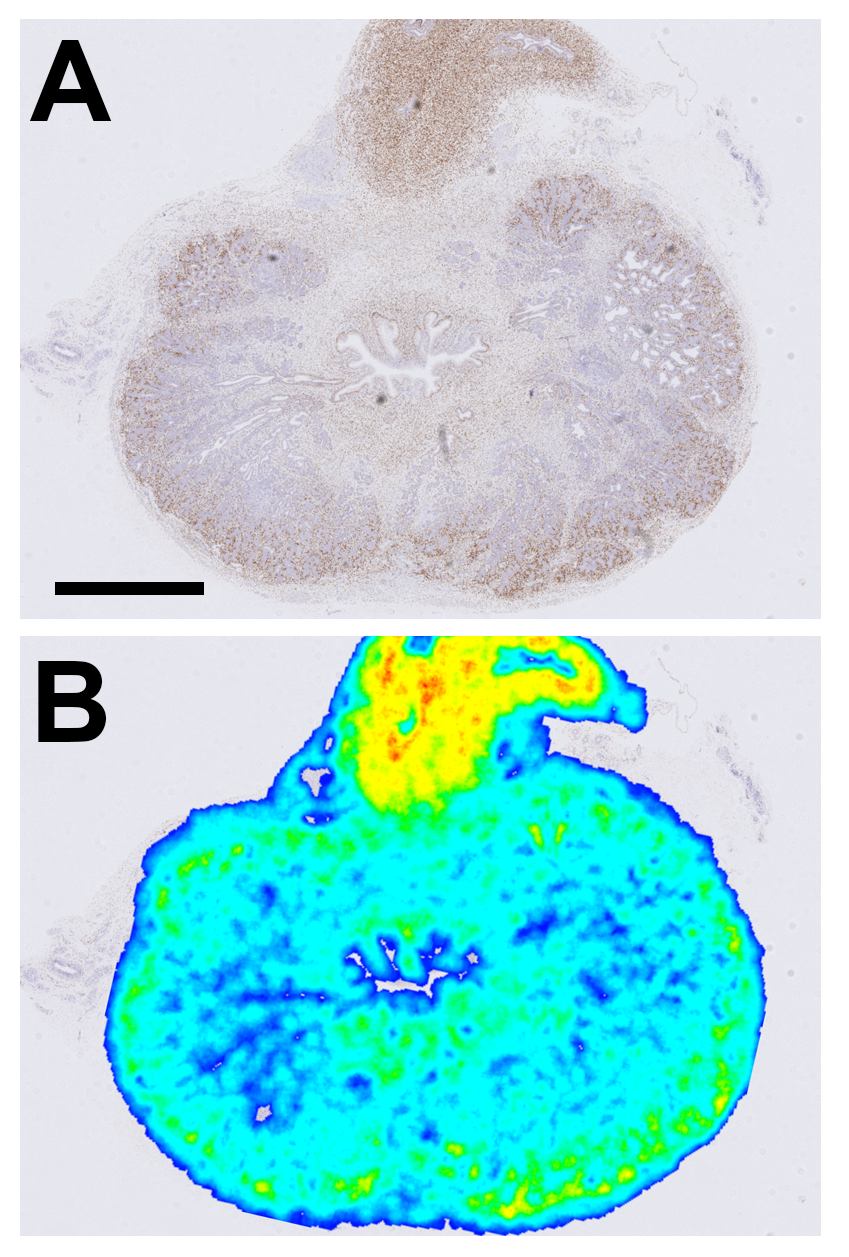

Supplement: Supplementary file 3 — Supporting Figure S3: Microphotograph of a premature canine prostate of a 2‐week old puppy, case 18. [file PROS-86-568-s001.tif]

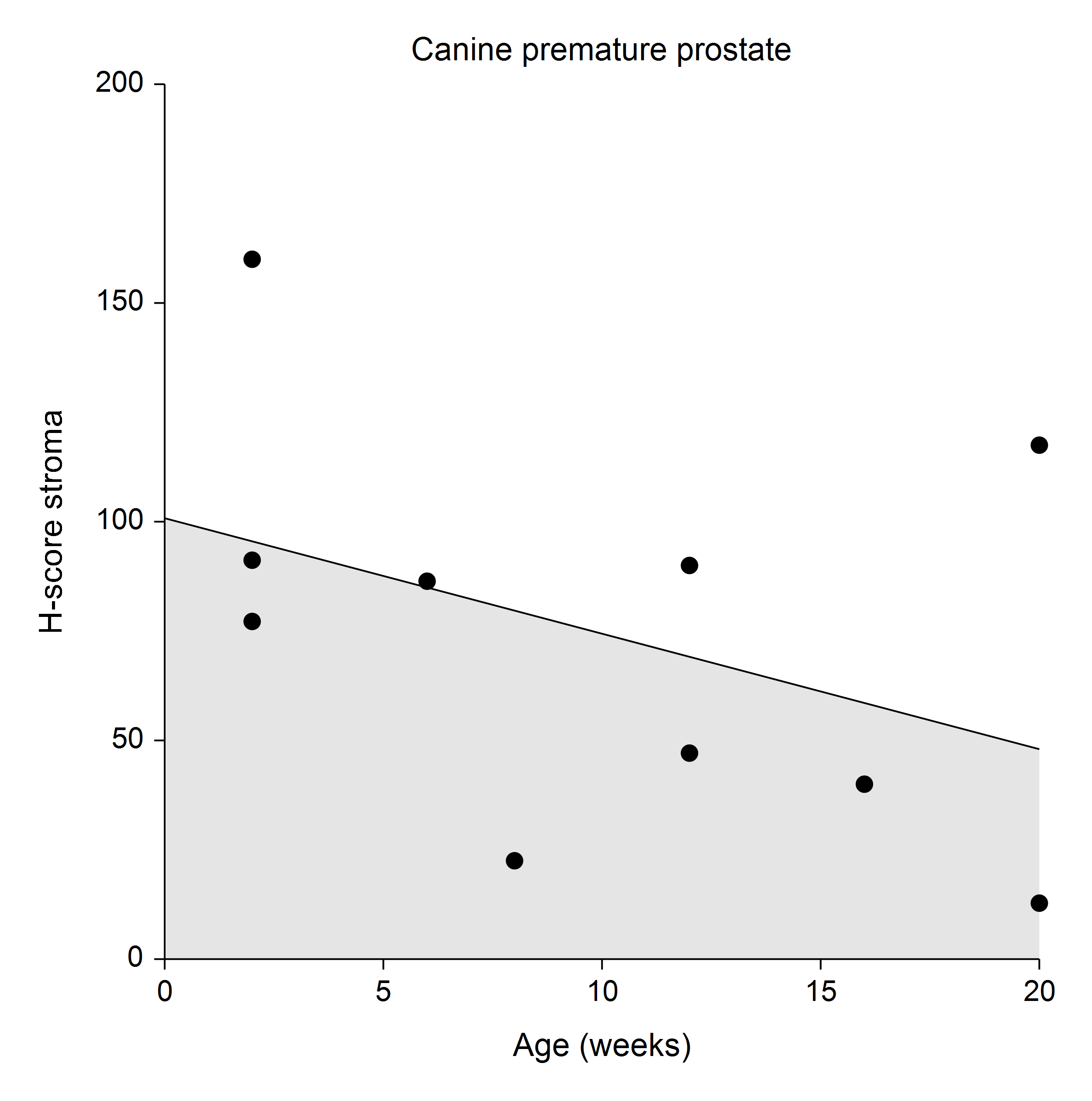

Supplement: Supplementary file 4 — Supporting Figure S4: Scatter plot illustrating the correlation between age and stromal ERα expression (H‐score) in canine premature prostate tissue. [file PROS-86-568-s002.tif]
